# Iterative Regression Based Hybrid Localization for Wireless Sensor Networks

**DOI:** 10.3390/s21010257

**Published:** 2021-01-02

**Authors:** Kyunghyun Lee, Sangkyeum Kim, Kwanho You

**Affiliations:** Department of Electrical and Computer Engineering, Sungkyunkwan University, Suwon 16419, Korea; naman2001@skku.edu (K.L.); interpost94@skku.edu (S.K.)

**Keywords:** time difference or arrival, angle of arrival, iteratively reweighted-recursive least squares, localization

## Abstract

Among various localization methods, a localization method that uses a radio frequency signal-based wireless sensor network has been widely applied due to its robustness against noise factors and few limits on installation location. In this paper, we focus on an iterative localization scheme for a mobile with a limited number of time difference of arrival (TDOA) and angle of arrival (AOA) data measured from base stations. To acquire the optimal location of a mobile, we propose a recursive solution for localization using an iteratively reweighted-recursive least squares (IR-RLS) algorithm. The proposed IR-RLS scheme can obtain the optimal solution with a fast computational speed when additional TDOA and/or AOA data is measured from base stations. Moreover, while the number of measured TDOA/AOA data was limited, the proposed IR-RLS scheme could obtain the precise location of a mobile. The performance of the proposed IR-RLS method is confirmed through some simulation results.

## 1. Introduction

Recently, the localization method to estimate a mobile location has attracted much attention with the growth of location-based service (LBS) industries, such as augmented reality, autonomous navigation systems, and the internet of things (IoT). The most widely used method for localization is the global positioning system (GPS), which is a satellite based radio-navigation system. However, GPS shows a performance distinction in the concrete jungle or indoor environment since the obstacles between a target and a satellite cause the time delay of satellite signal arrival. Utilization of satellite signals for localization cannot satisfy the localization accuracy that is required in recent LBS industry especially under indoor environment [1,2,3]. Among various localization methods for indoor environment application, localization using measurements associated with radio signals received at a base station has been applied to location-based industries due to its spatial usability and sufficient accuracy. Potential installation locations of wireless sensor networks that use radio frequency signals are relatively unrestricted compared to other localization methods like global positioning system. In addition, the propagation signal is less affected by noise factors since sensor networks that use radio frequency signals are typically used for near-field communication. For this reason, localization using radio frequency signals is robust to disturbance caused by interference and jamming in comparison with satellite signal-based localization [4,5,6,7].

The precise localization of a mobile with a limited number of measurement data is one of the most challenging and crucial issues in the location-based service. While radio signal-based localization demonstrates the better location estimation accuracy than other localization methods under the spatial constraints, such as densely developed urban areas with high-rise buildings, improved localization performance has been constantly required by location-based industries [8,9,10]. Environmental noise caused by external factors, such as ununiformed temperature, obstacles, and signal interference, can lead to estimation inaccuracy of a mobile location [11,12]. To improve localization accuracy, Jung [13] proposed an optical wireless indoor localization system using light emitting diodes. The typical detection areas of the system led to spatial diversity that prevented multipath fading under line-of-sight conditions. Zhang [14] suggested a self-built ultrasonic transmitter and an innovative base station-based indoor localization system using time difference of arrival (TDOA) measurements. Zhang applied the extended Kalman filter estimator to observe the state of a mobile by linearizing measurement equations. To locate the unmanned aerial vehicle (UAV) avoiding the nonline-of-sight (NLOS) bias error, Xu [15] proposed an efficient method using only three passive RF receivers. After decomposing the NLOS link into two line-of-sight (LOS) paths, the UAV position was estimated using the position of one-bounce scatters. Wang [16] induced a unified solution with a single model to locate the source for the hybrid angle of arrival (AOA) and TDOA positioning. Using Bhattacharyya-Barankin bound and iterative maximum likelihood estimator, a unified localization solution was derived without requiring the knowledge of the source’s closeness to the sensors. Cheung [17] suggested a unified constrained least squares (CLS)-based mobile localization approach for time of arrival (TOA), received signal strength (RSS), TDOA, AOA, and hybrid TDOA/AOA measurements. The linear geolocation equations were derived by reorganizing the nonlinear equations obtained from the measurements. The derived linear equations were solved in an optimum manner with the use of Lagrange multipliers method.

In this paper, we propose an iteratively reweighted-recursive least squares (IR-RLS) algorithm to improve the estimation accuracy of hybrid TDOA and AOA localization. The measured TDOA and AOA data are identified using Kalman filter-based hypothesis test whether the data is under LOS or NLOS environment. To improve the estimation of a mobile location, the identified LOS data is used for hybrid TDOA/AOA localization. The hybrid localization method using TDOA and AOA measurements can improve localization performance and reduce the minimum number of required base stations [18,19]. The IR-RLS method is a recursive solution of the weighted least squares (WLS) method with an $L_{p}$-norm-based weight matrix. In wireless sensor network-based localization, the additional data measured from other base stations can be received during or after computation to obtain a mobile location. With the WLS method, each process needs to be recomputed using additionally measured TDOA and/or AOA data. However, the IR-RLS method can obtain the optimal solution using a previous data-based solution and the additional measurement data. The IR-RLS method provides fast computational rate when additional data are received. Moreover, the covariance matrix can be obtained to denote the weights of each TDOA and AOA measurements through iteration processes using the IR-RLS method. The optimal solution with iteratively reweighted measurement data can improve the localization accuracy using a limited number of TDOA and AOA data from base stations.

The remainder of this paper is organized as follows. In Section 2, Kalman filter-based NLOS data identification method is introduced. Moreover, we derive a hybrid TDOA/AOA localization model that is based on separately derived TDOA and AOA equations. The iteration procedure to acquire the weights of each measurement using the $L_{p}$-norm approximation is presented in Section 3. Moreover, the recursive solution of IR-RLS method is derived to handle additional measurement data received from base stations. In Section 4, simulation results that show the effectiveness and efficiency of the proposed IR-RLS method are provided. Finally, conclusions are provided in Section 5.

## 2. TDOA/AOA Measurement-Based Localization Model

### 2.1. NLOS Data Identification Based on Kalman Filter

In wireless sensor network-based localization methods, NLOS problem can occur by the attenuation factors such as structures and walls between a mobile and base station. NLOS data should be discriminated for the improvement of the localization performance as NLOS problem causes the significant errors in measurements of TDOA and AOA data. To discriminate NLOS measurement data, at least one base station is assumed to be under LOS environment. The distance and velocity data calculated from the measured TDOA data at each base station are divided into several data groups depending on sampling time interval. With the definition of $r_{i}$ and $v_{i}$ as the distance and velocity between a mobile and a base station at the *i*-th sampling time, we can derive the state model for the *x*- and *y*-coordinates independently as a following equation [20].

(1) $$s_{i+1}={\mathrm{Gs}}_{i}+hn_{i},i=1,\cdots ,P,$$

where

$$G=\left[\begin{array}{cc} 1 & \Delta t\\ 0 & 1 \end{array}\right],h=\left[\begin{array}{c} 0\\ \Delta t \end{array}\right].$$

In Equation (1), state vector $\left[{s_{i}}_{T}\right]$ represents ${\left[r_{i}\;v_{i}\right]}^{T}$. $n_{i}$ is a driving noise variable with a variance $Q_{s}=\rho_{s}^{2}I$. The measured distance data ($z_{i}$) between a mobile and a base station can be acquired as follows:

(2) $$z_{i+1}={\mathrm{Ds}}_{i}+m_{i},$$

with $D=\left[1\;0\right]$. $m_{i}$ is the environmental noise value with a covariance of $q_{z}=\rho_{z}^{2}$. The iterative process of Kalman filter for state estimation can be derived using Equations (1) and (2) as follows:

(3) $$\begin{array}{ccc} {\hat{s}}_{i}^{-} & = & G{\hat{s}}_{i-1},\;\\ P_{i}^{-} & = & {\mathrm{GP}}_{i-1}G^{T}+{\mathrm{hQ}}_{s}h^{T},\;\\ K_{i} & = & P_{i}^{-}D^{T}{\left({\mathrm{DP}}_{i}^{-}D^{T}+q_{z}\right)}^{-1},\;\\ {\hat{s}}_{i} & = & {\hat{s}}_{i}^{-}+K_{i}\left(z_{i}-D{\hat{s}}_{i}^{-}\right),\;\\ P_{i} & = & P_{i}^{-}-K_{i}{\mathrm{DP}}_{i}^{-}, \end{array}$$

where $P_{i}$ denotes the covariance matrix of a state vector $s_{i}$. The estimated state ${\hat{s}}_{i}$ and the covariance $P_{i}$ are valid for Kalman gain $K_{i}$. Using Kalman filter based state estimation process, denoted by Equation (3), the distance between a mobile and each base station can be estimated.

On the basis of the estimated distance at the sampling time $t_{j}$, denoted by $r_{Kalman}\left(t_{j}\right)$, the standard deviation of the computed distance $r_{m}\left(t_{j}\right)$ can be obtained as follows:

(4) $$\hat{\sigma }=\sqrt{\frac{1}{N}\sum_{j=1}^{N}{\left[r_{m}\left(t_{j}\right)-r_{Kalman}\left(t_{j}\right)\right]}^{2}},$$

where the parameter *N* is the number of distance data in each data group. The standard deviation of LOS data (${\hat{\sigma }}_{LOS}$) can be computed using Equation (4). Then, the data group contaminated by NLOS noise can be identified using the following hypothesis test with the threshold parameter τ that can be determined according to the environment.

(5) $$\begin{array}{ccc} H_{1}\left(LOS\;condition\right) & : & \hat{\sigma }<\tau {\hat{\sigma }}_{LOS},\;\\ H_{2}\left(NLOS\;condition\right) & : & \hat{\sigma }\geq \tau {\hat{\sigma }}_{LOS}. \end{array}$$

In the next section, the localization model is obtained using LOS TDOA/AOA data identified by Equation (5).

### 2.2. Hybrid TDOA/AOA Localization Model

The localization performance can be improved by combining different localization methods. Moreover, the number of base stations required for localization can be reduced by employing a hybrid method [21,22]. Among various localization methods that use radio frequency signals, the widely used data for mobile localization are TDOA and AOA measurements. In this paper, a hybrid localization model that uses TDOA/AOA measurements is considered. The TDOA method uses the difference between signal arrival times at a mobile and each base station at a known location [23,24]. The AOA method is the localization using the determined direction of radio signal propagation on an antenna array. AOA measurements can be computed by measuring the TDOA at individual elements of the array [25]. In two-dimensional spaces, more than two base stations that measure both TDOA and AOA data are required to estimate a mobile location [26].

To formulate a TDOA/AOA measurements-based hybrid localization model, we derive the relationship between a mobile location and each measurement data in this section. Consider that one mobile and *M* base stations whose locations are $m={\left[x\;y\right]}^{T}$ and $b_{k}={\left[x_{k}\;y_{k}\right]}^{T}$, respectively, are distributed over a two-dimensional surface without loss of generality. With given M−1 TDOA data, the unknown location of a mobile can be obtained as follows:

(6) $$\left(x_{k}-x_{1}\right)\left(x-x_{1}\right)+\left(y_{k}-y_{1}\right)\left(y-y_{1}\right)+r_{k1}^{0}r_{1}^{0}=\frac{1}{2}\left[{\left(x_{k}-x_{1}\right)}^{2}+{\left(y_{k}-y_{1}\right)}^{2}-{\left(r_{k1}^{0}\right)}^{2}\right],$$

where $r_{k}^{0}$ is the distance between a mobile and the *k*-th base station. $r_{k1}^{0}$ denotes the range difference between $r_{k}^{0}$ and $r_{1}^{0}$. Equation (6) can be presented in the following matrix form.

(7) $$B_{TDOA}u=w_{TDOA},$$

where

$$\begin{array}{ccc} B_{TDOA} & = & \left[\begin{array}{ccc} x_{2}-x_{1} & y_{2}-y_{1} & r_{21}^{o}\\ \vdots & \vdots & \vdots \\ x_{M}-x_{1} & y_{M}-y_{1} & r_{M1}^{o} \end{array}\right],\;\\ w_{TDOA} & = & \left[\begin{array}{c} \frac{1}{2}\left({\left(x_{2}-x_{1}\right)}^{2}+{\left(y_{2}-y_{1}\right)}^{2}-{\left(r_{21}^{o}\right)}^{2}\right)\\ \vdots \\ \frac{1}{2}\left({\left(x_{M}-x_{1}\right)}^{2}+{\left(y_{M}-y_{1}\right)}^{2}-{\left(r_{M1}^{o}\right)}^{2}\right) \end{array}\right]. \end{array}$$

In Equation (7), $u={\left[x-x_{1}\;y-y_{1}\;r_{1}^{0}\right]}^{T}$ contains the *x*- and *y*-locations of a mobile. The AOA of the transmitted signal from the mobile to the *k*-th base station, denoted by $\varphi_{k}$, can be represented by the relationship between the *x*- and *y*-locations of a mobile and each base station.

(8) $$\tan\left(\varphi_{k}\right)=\frac{\sin(\varphi_{k})}{\cos(\varphi_{k})}=\frac{y-y_{k}}{x-x_{k}}.$$

Equation (8) can be rewritten as follows:

(9) $$x\sin\left(\varphi_{k}\right)-y\cos\left(\varphi_{k}\right)=x_{k}\sin\left(\varphi_{k}\right)-y_{k}\cos\left(\varphi_{k}\right).$$

The AOA localization Equation (9) can be rewritten as the following matrix form.

(10) $$A_{AOA}v=w_{AOA},$$

where

$$\begin{array}{ccc} A_{AOA} & = & \left[\begin{array}{cc} \sin(\varphi_{1}) & -\cos(\varphi_{1})\\ \vdots & \vdots \\ \sin(\varphi_{M}) & -\cos(\varphi_{M}) \end{array}\right],\;\\ w_{AOA} & = & \left[\begin{array}{c} 0\\ \left(x_{2}-x_{1}\right)\sin\left(\varphi_{2}\right)-\left(y_{2}-y_{1}\right)\cos\left(\varphi_{2}\right)\\ \vdots \\ \left(x_{M}-x_{1}\right)\sin\left(\varphi_{M}\right)-\left(y_{M}-y_{1}\right)\cos\left(\varphi_{M}\right) \end{array}\right], \end{array}$$

with $v={\left[x-x_{1}\;y-y_{1}\right]}^{T}$. While there are only two base stations from which AOA data can be measured, the *x*- and *y*-locations of a mobile can be acquired from the variable v in Equation (10). Combining different measurements of the received radio signals in wireless sensor network can enhance localization performance and reduce the required number of base stations [27,28,29]. The hybrid localization equation using TDOA/AOA data can be expressed in linear matrix form using Equations (7) and (10).

(11) Bu=w,

where

$$B=\left[\begin{array}{c} B_{TDOA}\\ B_{AOA} \end{array}\right],w=\left[\begin{array}{c} w_{TDOA}\\ w_{AOA} \end{array}\right].$$

Here, $B_{AOA}$ is defined as $\left[A_{AOA}\;0\right]$.

## 3. Optimal Solution Using IR-RLS Scheme

To obtain the optimal solution to the localization formula of Equation (11), the determination of the covariance matrix that denotes the weight of each TDOA/AOA data is a significant factor. In this section, we suggest an IR-RLS scheme that obtains a recursive solution with an iteratively reweighted covariance matrix of each measured TDOA and AOA data. The IR-RLS scheme-based regression method can obtain the optimal solution through the covariance matrix that uses the $L_{p}$-norm approximation of an estimation error.

To minimize the estimation error of each row vector in Equation (11) with *M* base stations, the localization objective function can be formulated as follows:

(12) $$\arg\min\sum_{i=1}^{2M-1}{\left|w_{i}-b_{i}\hat{u}\right|}^{p},$$

where $w_{i}$ and $b_{i}$ denote the *i*-th measurement scalar parameter of w and the raw vector of B in Equation (11), respectively. In general, the covariance matrix of the WLS can be acquired using the Euclidean norm [30]. The scalar error of estimation can be derived through the covariance matrix of the WLS as follows:

(13) $$\varepsilon_{WLS}=\sum_{i=1}^{2M-1}c_{i}^{2}{\left|w_{i}-b_{i}\hat{u}\right|}^{2}=e^{T}C^{T}Ce,$$

where $e=B\hat{u}-w$. In Equation (13), $C=diag\left\{c_{1},\cdots ,c_{2M-1}\right\}$ is a weight matrix. The WLS solution of the *j*-th iteration can be derived using the scalar error of the (j−1)-th iteration result. Using Equations (12) and (13), C(j) can be derived as follows:

(14) $$\begin{array}{ccc} C(j) & = & diag\left\{c_{1}\left(j\right),\cdots ,c_{2M-1}\left(j\right)\right\},\;\\ c_{i}\left(j\right) & = & {\left|w_{i}-b_{i}\hat{u}\right|}^{\frac{p-2}{2}}, \end{array}$$

where $\hat{u}$ denotes the optimal solution of the (j−1)-th iteration. Using a reweighted covariance variable C(j), the optimal solution of the *j*-th iteration can be acquired as follows:

(15) $$\hat{u}\left(j\right)={\left[B^{T}C^{T}\left(j\right)C\left(j\right)B\right]}^{-1}B^{T}C^{T}\left(j\right)C\left(j\right)w.$$

We begin the first iteration with a weight matrix C(1) as the identity matrix [31]. Based on Equation (15), with (i+1) measurement data obtained from radio frequency signal of the additional base stations, the hybrid TDOA/AOA localization equation can be rewritten as follow:

(16) $$\left[\begin{array}{c} B_{i}\\ b_{i+1} \end{array}\right]u=\left[\begin{array}{c} w_{i}\\ w_{i+1} \end{array}\right],$$

where $B_{i}={\left[b_{1}\;\cdots \;b_{i}\right]}^{T}$ expresses the matrix that includes *i* data. The vector $w_{i}$ represents ${\left[w_{1}\;\cdots \;w_{i}\right]}^{T}$. $b_{i+1}$ and $w_{i+1}$ represent the (i+1)-th measurement raw vector and a scalar parameter, respectively. The WLS solution using (i+1) measurement data and the *j*-th iterated weight matrix $C_{i+1}\left(j\right)$, denoted by ${\hat{u}}_{i+1}$ for notational simplicity, can be rewritten as follows:

(17) $${\hat{u}}_{i+1}={\left[B_{i+1}^{T}Q_{i+1}B_{i+1}\right]}^{-1}B_{i+1}^{T}Q_{i+1}w_{i+1},$$

where $Q_{i+1}=diag\left\{q_{1}\cdots q_{i+1}\right\}$ represents $C_{i+1}^{T}\left(j\right)C_{i+1}\left(j\right)$.

With the definition of $Z_{i+1}={\left[B_{i+1}^{T}Q_{i+1}B_{i+1}\right]}^{-1}$, the following equation can be derived:

(18) $$Z_{i+1}^{-1}=Z_{i}^{-1}+b_{i+1}^{T}Q_{i+1}\left(j\right)b_{i+1}.$$

Using Equation (18), the WLS solution can be rewritten as follows:

(19) $$\begin{array}{ccc} {\hat{u}}_{i+1} & = & Z_{i+1}\left(B_{i}^{T}Q_{i}w_{i}+b_{i+1}^{T}q_{i+1}w_{i+1}\right)\;\\ \; & = & Z_{i+1}\left(Z_{i}^{-1}Z_{i}B_{i}^{T}Q_{i}w_{i}+b_{i+1}^{T}q_{i+1}w_{i+1}\right). \end{array}$$

Since ${\hat{u}}_{i}=Z_{i}B_{i}Q_{i}w_{i}$, Equation (19) can be rewritten as follows:

(20) $$\begin{array}{ccc} {\hat{u}}_{i+1} & = & Z_{i+1}\left(Z_{i}^{-1}{\hat{u}}_{i}+b_{i+1}^{T}q_{i+1}w_{i+1}\right)\;\\ \; & = & {\hat{u}}_{i}+Z_{i+1}b_{i+1}^{T}q_{i+1}\left(w_{i+1}-b_{i+1}{\hat{u}}_{i}\right). \end{array}$$

Using $Z_{i+1}={\left(B_{i}^{T}Q_{i}B_{i}+b_{i+1}^{T}q_{i+1}b_{i+1}\right)}^{-1}$, the IR-RLS solution can be derived as follows:

(21) $${\hat{u}}_{i+1}={\hat{u}}_{i}+{\left[B_{i}^{T}C_{i}^{T}\left(j\right)C_{i}\left(j\right)B_{i}+b_{i+1}^{T}c_{i+1}^{2}\left(1\right)b_{i+1}\right]}^{-1}b_{i+1}^{T}c_{i+1}^{2}\left(1\right)\left(w_{i+1}-b_{i+1}{\hat{u}}_{i}\right).$$

**Figure 1:**
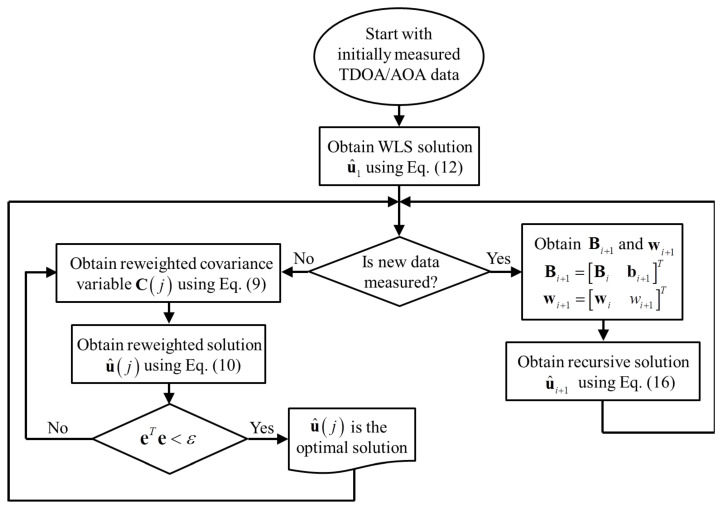
Flowchart of the iteratively reweighted-recursive least squares (IR-RLS) procedure.

In Equation (21), the initial weight of the (i+1)-th measurement data, denoted by $c_{i+1}\left(1\right)$, should be set to 1 if the auxiliary method to determine the covariance matrix is not applied. The IR-RLS solution using (i+1) TDOA and AOA measurements can be obtained with the computed values using *i* measured data (${\hat{u}}_{i}$, $B_{i}$, $C_{i}\left(j\right)$, and $w_{i}$) and (i+1)-th measurement data ($b_{i+1}$, $c_{i+1}\left(1\right)$, and $w_{i+1}$). To compute the weight of (i+1)-th measurement data, the iteration process using Equation (14) can be carried out with ${\hat{u}}_{i+1}$ and $C_{i+1}\left(1\right)$. The proposed IR-RLS scheme can obtain the solution with relatively fast computational speed. Moreover, although the number of measured TDOA/AOA data was limited, the precise location of a mobile can be obtained through the $L_{p}$-norm-based iteration process of a covariance matrix in the proposed IR-RLS scheme. The overall process of the proposed IR-RLS scheme is demonstrated in Algorithm 1. Following the procedures in Algorithm 1, the flowchart of the recursive optimization process is shown in Figure 1.
**Algorithm 1** IR-RLS scheme**Input:** Measurement data ($r_{i1}$, $\varphi_{i}$), error threshold (ε),          norm value (*p*)**Output:** Optimal solution ($\hat{u}$)  1: **Initialization** Obtain ${\hat{u}}_{1}$ using Equation (17)   2: **while**
*new data is measured***do**  3:      Obtain ${\hat{u}}_{i}$ using Equation (21)  4:      **while**
$e^{T}e\geq \varepsilon$**do**   5:            Obtain $C_{i}\left(j\right)$ using Equation (14)  6:            Obtain ${\hat{u}}_{i}\left(j\right)$ using Equation (15)   7:            j=j+1   8:      **end while**  9:      $B_{i+1}={\left[B_{i}\;b_{i+1}\right]}^{T}$10:      $w_{i+1}={\left[w_{i}\;w_{i+1}\right]}^{T}$ 11:      i=i+1 12: **end while**

## 4. Simulations

In Section 4, the effectiveness of the proposed IR-RLS-based localization scheme applied to TDOA/AOA model is verified via simulations. The radio frequency signal from a mobile is assumed to be measured by five base stations positioned at (0, 0), (0, 50), (0, 100), (100, 0), and (100, 50) m. The environmental and NLOS noises in TDOA and AOA data are assumed to follow the Gaussian distribution. Variances of TDOA and AOA environmental noises are set as $8.33\times 10^{-9}$ s and $0.15^{\circ }$, respectively. Therefore, the variance of range difference parameter ($r_{k1}^{0}$) is 2.5 m with a signal propagation speed of $3\times 10^{8}$ m/s. As NLOS noise causes a significant transmission time delay and incident angle change in TDOA and AOA measurements, the variances of NLOS noises are set as ten times greater than the variances of environmental noises. Moreover, we set hypothesis test parameter τ, norm value *p*, and iteration parameter ε as 5, 2.5, and 0.1, respectively. The simulated trajectory has 200 time samples at one second intervals.

**Figure 2:**
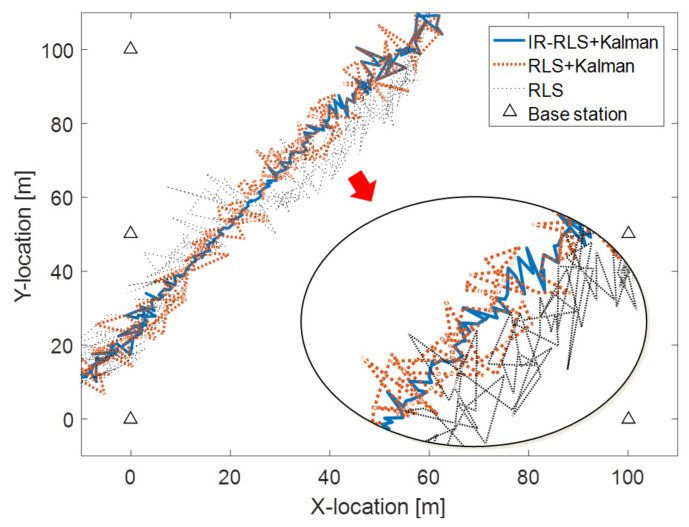
Estimated trajectory comparison between iteratively reweighted- recursive least squares (IR-RLS) and RLS methods.

**Figure 3:**
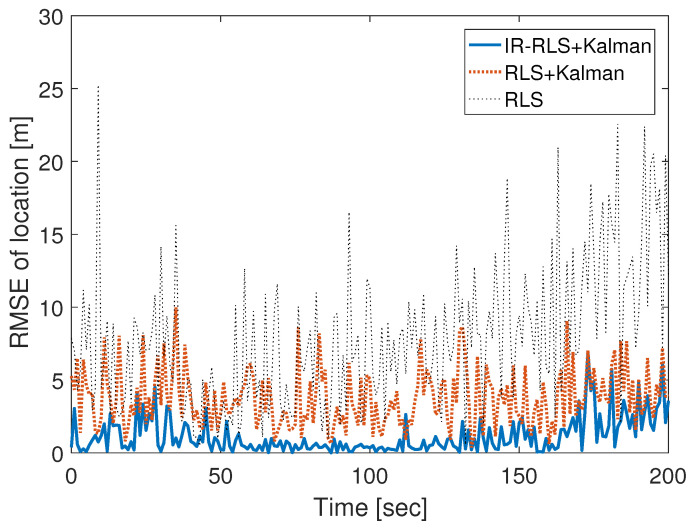
Root mean square error (RMSE) comparison between IR-RLS and RLS methods.

The comparison of estimated trajectories using the recursive least squares (RLS) and IR-RLS algorithms is shown in Figure 2. RLS method is an adaptive algorithm that finds iteratively the optimal coefficients to minimize a linear least squares cost function. In RLS method, all the weights of TDOA and AOA data are exactly the same as the covariance matrix is an identity matrix. To confirm the performance of the proposed NLOS data identification test based on Kalman filter, RLS method is applied to both the unclassified TDOA/AOA data and the identified LOS data-based localization models. In Figure 2, the mobile moves in straight line from (0, 10) m to (60, 110) m. In the simulation, signal attenuation happens between a mobile transferring from (0, 10) m to (30, 60) and a base station on (0, 50) m. Moreover, there is attenuation between a mobile transferring from (30, 60) m to (60, 110) m and a base station on (0, 100) m. In Figure 2, the acquired trajectory of a mobile using the IR-RLS algorithm is denoted by the thick solid line. The estimated trajectories using the unclassified data and the identified LOS data through RLS algorithm are represented by the thin dotted line and thick dotted line, respectively. As shown in Figure 2, the localization result driven by Kalman filter hypothesis test-based LOS data denotes better estimation performance than the estimated trajectory using the unclassified data. Moreover, the estimated trajectory obtained using the IR-RLS algorithm follows the real trajectory more closely than the estimated trajectory obtained by the RLS algorithm. Root mean square error (RMSE) results comparison between IR-RLS and RLS estimations using LOS TDOA/AOA data identified by Kalman filter is demonstrated as thick solid and dotted lines in Figure 3, respectively. RMSE of RLS result using the unclassified data is denoted as a thin dotted line. As shown in Figure 3, the RMSE of the estimated mobile location using the IR-RLS scheme is much less than the RMSE driven by the RLS method since the covariance matrix obtained through the $L_{p}$-norm-based iteration process determines effectively the weight of each TDOA/AOA data. The proposed NLOS identification and IR-RLS methods show the performances with the lowest RMSE result in Figure 3.

**Figure 4:**
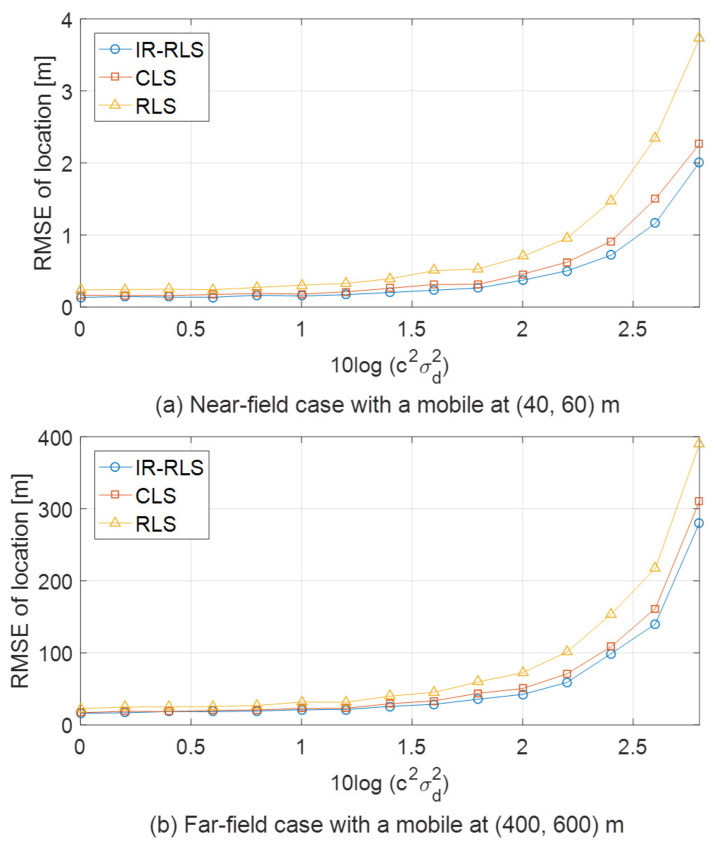
Estimation comparison for near-field and far-field mobile cases.

**Figure 5:**
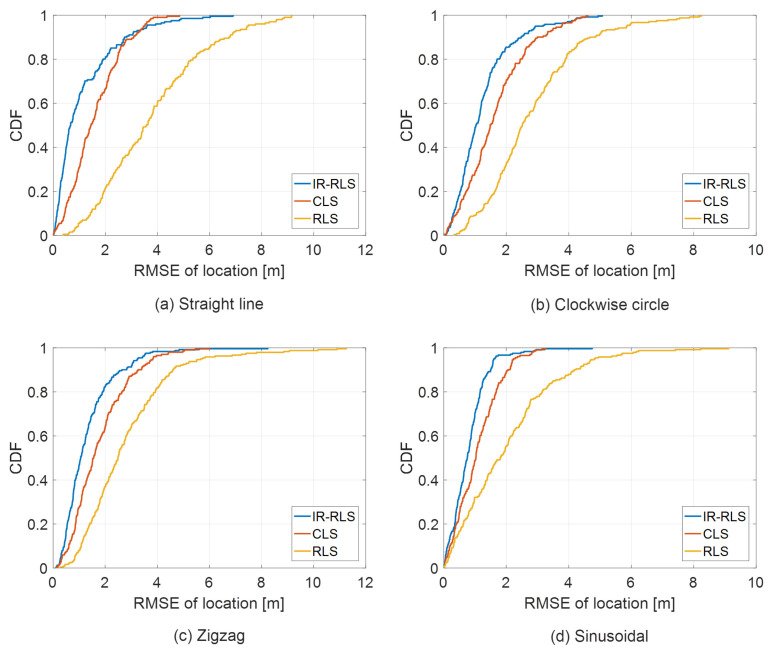
Cumulative distribution function (CDF) comparison of RMSE for different trajectory cases.

Figure 4 shows RMSE results for IR-RLS, CLS, and RLS methods for different mobile and base station layouts. CLS method in which the Lagrange multiplier is applied to TDOA/AOA localization model-based objective function with a constraint was proposed in [17]. The constraint represents the relationship between a mobile location and TOA measurement from the first base station. In Figure 4, the upper and lower graphs show RMSE results for a near-field mobile at (40, 60) m and a far-field mobile at (400, 600) m, respectively. Circle, square, and triangle marked lines in each plot denote RMSE results using IR-RLS, CLS, and RLS methods, respectively. The proposed IR-RLS method based on iteratively reweighted measurement data shows more precise estimation performance compared to the RLS algorithm for both near-field and far-field cases as shown in Figure 4. Moreover, the proposed method has improved performance than CLS and RLS-based estimation in every noise environment with a gentle slope for both near-field and far-field cases. The cumulative distribution function (CDF) error plots of the estimated trajectories using IR-RLS, CLS, and RLS algorithms are shown in Figure 5. The mobiles in Figure 5a–d travel in straight line, clockwise circle, zigzag, and sinusoidal routes, respectively. For each trajectory case in Figure 5, the proposed IR-RLS method outperforms compared with CLS and RLS methods due to iteratively reweighted procedure. Moreover, Table 1 shows the mean, median, minimum, and maximum values of RMSE for each trajectory case derived from the results of Figure 5. We can confirm that mean and median values of RMSE using IR-RLS algorithm are the lowest compared with other results in each trajectory case. On the contrary, the results of RLS method show the worst performances in every trajectory case as there are no covariance iteration and constraint for RLS-based localization.

**Figure 6:**
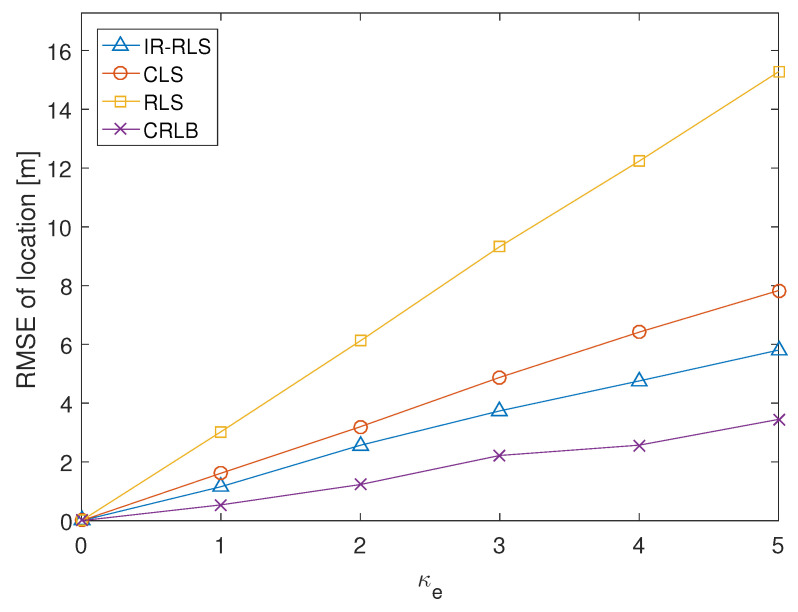
RMSE comparison for proportional noise parameter ($\kappa_{e}$).

Figure 6 depicts the performance comparison among the results of IR-RLS, CLS, and RLS algorithms. Figure 6 demonstrates the RMSE of three algorithms versus the increase of TDOA and AOA environmental noise. We increase the absolute noise values in TDOA and AOA measurements using a proportional noise parameter $\kappa_{e}$ to prove the RMSE performance according to the noise magnitude variation. $\kappa_{e}$ is the coefficient multiplied to the environmental noise variables in TDOA and AOA data ($r_{k1}^{0}$ and $\varphi_{k}$). The Cramer-Rao lower bound (CRLB) is also demonstrated as a reference. As shown in Figure 6, the RMSE of the proposed IR-RLS result is smaller than the CLS and RLS results for the same magnitude of TDOA/AOA environmental noises.

The practical runtime of IR-RLS algorithm can change depending on the number of weight iteration. In this paper, the computational complexity of IR-RLS algorithm is confirmed through simulation as there is a limit to represent the exact computational cost of weight iteration using the complexity analysis such as floating point operations. In Table 2, the computational time and RMSE results of the estimated mobile’s location through least squares (LS), CLS, and IR-RLS methods are compared for different number of the base stations ($N_{b}$) that measure additional TDOA and AOA data. In the simulation, the base stations that measure additional data are located randomly in xy-plane of Figure 2. Each base station is assumed to measure one set of TDOA and AOA data. Both performances of IR-RLS using iterated weight matrix or conventional weight matrix are demonstrated in Table 2. As shown in Table 2, the solution using IR-RLS method with iterated weight matrix is obtained in less computational time compared with the result of CLS method. Since the procedure to solve the equations for Lagrange multiplier of CLS solution demands huge computational complexity, the solution of CLS method takes more time than other methods. While the proposed IR-RLS algorithm with weight iteration shows more computational time than LS method due to the calculation of weight matrix update, the computational speed of IR-RLS with conventional weight matrix is faster than LS method. Therefore, the proposed recursive solution of IR-RLS algorithm confirms the computational efficiency. Moreover, the proposed IR-RLS solution shows a good accuracy performance relatively compared to other methods for different base station numbers.

## 5. Conclusions

In this paper, we proposed a hybrid TDOA/AOA localization method to obtain the precise mobile location using an IR-RLS algorithm. Since both TDOA and AOA measurements are relatively robust against disturbances caused by interference and jamming, the TDOA/AOA hybrid localization method can improve localization performance and decrease the required number of base stations. The IR-RLS algorithm provided fast computation when additional TDOA or AOA data was received. Therefore, the IR-RLS method can handle more measurement data compared to the WLS method. Moreover, the IR-RLS scheme iteratively obtained the $L_{p}$-norm approximation-based covariance matrix using the estimation error of the previous iteration. While the number of measured TDOA/AOA data was limited, the precise location of a mobile can be obtained through the proposed IR-RLS scheme. The simulation results confirmed the effectiveness of the IR-RLS scheme.

## Figures and Tables

**Table 1:** Mean, median, minimum, and maximum of RMSE in each trajectory case.

Method	Trajectory	Mean	Median	Min	Max
**IR-RLS**	Straight line	1.153	0.664	0.008	6.902
	Clockwise circle	1.243	1.029	0.011	5.075
	Zigzag	1.331	1.062	0.115	4.752
	Sinusoidal	0.806	0.752	0.002	4.752
**CLS**	Straight line	1.599	1.443	0.002	4.846
	Clockwise circle	1.641	1.484	0.025	4.610
	Zigzag	1.777	1.553	0.131	5.984
	Sinusoidal	1.084	1.029	0.002	3.261
**RLS**	Straight line	3.778	3.579	0.379	9.160
	Clockwise circle	2.817	2.514	0.332	8.246
	Zigzag	2.804	2.490	0.272	11.258
	Sinusoidal	2.042	1.811	0.003	9.116

**Table 2:** RMSE and computational time comparison for different base station numbers.

Algorithm		$N_{b}=3$	$N_{b}=5$	$N_{b}=10$	$N_{b}=20$
**LS**	RMSE [m]	3.724	3.620	3.598	3.575
	Time [s]	0.740	0.825	1.714	2.890
**CLS**	RMSE [m]	1.785	1.653	1.614	1.593
	Time [s]	2.663	3.196	5.590	8.887
**IR-RLS w/conventional weight**	RMSE [m]	2.941	2.792	2.678	2.620
	Time [s]	0.654	0.731	1.321	2.183
**IR-RLS w/weight iterations**	RMSE [m]	1.529	1.521	1.476	1.459
	Time [s]	1.227	1.580	2.676	3.921

## Data Availability

Data sharing not applicable.
